# The Impact of Radiotherapy on the Outcomes of Deep Inferior Epigastric Artery Perforator Flaps for Breast Reconstruction: A Systematic Review and Meta-Analysis

**DOI:** 10.1007/s00266-026-05690-w

**Published:** 2026-03-11

**Authors:** Ali Mohamed Elameen, Ahmed Ibrahim Yassin

**Affiliations:** Department of Plastic and Reconstructive Surgery, El-Sahel Teaching Hospital, GOTHI, 2 Youssef Karam Street, Borham, El Sahel, Cairo Governorate Egypt

**Keywords:** Deep inferior epigastric artery perforator flap, Breast, Reconstruction, Radiotherapy

## Abstract

**Background:**

The deep inferior epigastric artery (DIEP) perforator flap is the primary reconstructive approach for autologous breast reconstruction. It is associated with minimal donor site complications and acceptable aesthetic outcomes for both the abdomen and breast. This meta-analysis evaluated the impact of radiotherapy on the DIEP flap for patients with autologous breast reconstruction.

**Methods:**

All clinical studies involving patients who underwent DIEP flap breast reconstruction and compared the outcomes of irradiated and non-irradiated breasts were included. A comprehensive literature search was performed across 12 databases up to May 18, 2025.

**Results:**

Eight studies including 4,447 patients (4478 flaps) were analyzed, of whom 1,624 received radiotherapy. Radiotherapy was associated with higher risks of partial flap loss (RR 1.69; 95% CI 0.99–2.90; *P* = 0.05) and wound revisions (RR 1.23; 95% CI 1.01–1.50; *P* = 0.04). Pre-DIEP radiotherapy significantly increased the risk of wound healing disturbances (RR 1.62; 95% CI 1.06–2.49; *P* = 0.03). No statistically significant differences were observed in flap volume change, total flap loss, flap contracture, fat necrosis, recipient-site infection, or the need for reconstructive adjustments. There was a significant lower total breast satisfaction score among irradiated breasts (MD − 6.49; 95% CI − 11.79 to − 1.19; *P* = 0.02).

**Conclusions:**

Radiotherapy adversely affects surgical and patient-reported outcomes following DIEP flap autologous breast reconstruction, with pre-DIEP radiotherapy significantly increasing the risk of wound healing disturbances and post-DIEP radiotherapy associated with reduced overall breast satisfaction.

**Level of Evidence I:**

This journal requires that authors assign a level of evidence to each article. For a full description of these Evidence-Based Medicine ratings, please refer to the Table of Contents or the online Instructions to Authors  www.springer.com/00266.

**Supplementary Information:**

The online version contains supplementary material available at 10.1007/s00266-026-05690-w.

## Introduction

Breast reconstruction is currently considered an integral part of breast cancer treatment. It is categorized into either autologous or implant-based reconstruction [1]. Although autologous breast reconstruction is a more complex surgical procedure, it is associated with more desirable aesthetic and psychological outcomes [2, 3]. Since the introduction of the deep inferior epigastric artery (DIEP) perforator flap, it has become the primary approach for autologous breast reconstruction, offering minimal donor site complications and acceptable aesthetic outcomes for both the abdomen and breast [4]. Breast reconstruction significantly enhances the quality of life for breast cancer survivors. Immediate breast reconstruction offers early breast mound recreation and reduces overall costs, resulting in satisfactory psychological and oncological outcomes [5]. As the number of patients seeking breast reconstruction increases, radiotherapy has become an integral component of breast cancer care. However, the consequences of radiotherapy in the context of autologous breast reconstruction pose considerable challenges. Radiotherapy improves locoregional control in patients at higher risk of breast cancer recurrence [6]. Conversely, radiotherapy can cause deleterious aesthetic consequences for the reconstructed breasts, including flap volume reduction, contracture, and reconstruction failure [7]. Irradiated breasts carry a substantial risk of infection, stromal atrophy, hyperpigmentation, flap fibrosis, and breast asymmetry, all of which compromise both oncological safety and aesthetic outcomes [8].

One of the main objectives of breast reconstruction is to achieve symmetrical volume restoration between breasts. Understanding the extent to which the volume of a DIEP flap will be retained after radiotherapy is crucial for preoperative planning to allow for overcompensation of flap loss after breast reconstruction [9]. The impact of radiotherapy on DIEP flap outcomes may considerably affect breast symmetry and lead to patient dissatisfaction [10]. Despite the therapeutic merits of radiotherapy, its impact on the aesthetic outcomes of breast reconstruction using a DIEP flap requires further evaluation. This is because of the desire of oncoplastic breast surgeons to achieve acceptable aesthetic results while preserving oncological safety. The integration of radiation therapy and breast reconstructive surgeries has not yet been comprehensively established. There is increased evidence supporting the negative impact of radiotherapy on the outcomes of autologous breast reconstruction. Therefore, it is crucial to recognize the extent of morbidity caused by radiotherapy on DIEP flaps in breast reconstruction [11, 12]. The literature is inconclusive regarding the impact of radiotherapy on the aesthetic and surgical outcomes of DIEP flaps and the identification of patients at higher risk of developing poor cosmetic results. This is due to considerable heterogeneity between previously published studies regarding reconstruction procedures, radiotherapy protocols, follow-up periods, and the scales implemented to assess aesthetic outcomes [13]. Furthermore, the majority of the published articles are non-comparable, with a lack of comprehensive evidence related to the cosmetic and surgical outcomes of DIEP flaps for breast reconstruction in the setting of radiotherapy. The morphological changes before or after radiotherapy need to be assessed to assist oncoplastic surgeons in precisely evaluating the long-term aesthetic outcomes of DIEP flaps for breast reconstruction.

This systematic review evaluates the impact of radiotherapy on the DIEP flap in patients undergoing autologous breast reconstruction. Such knowledge may reveal the challenges that arise when radiation therapy is delivered in the context of breast reconstruction. Identifying such evidence could help individualize the appropriate radiotherapy regimen and reconstructive approach, along with implementing refinement procedures to lessen the negative consequences of radiotherapy on the DIEP flap in breast reconstruction.

## Methods

This systematic review was conducted in accordance with the Preferred Reporting Items for Systematic Reviews and Meta-Analyses (PRISMA) guidelines [14], and the Cochrane collaboration recommendations [15] (Supplementary Table 1). The review protocol was prospectively registered in the PROSPERO database (registration number: CRD420251076611).

### Eligibility Criteria

All clinical studies that included patients who underwent DIEP flap breast reconstruction, with reported outcomes for both irradiated and non-irradiated breasts, were included. Studies were excluded if relevant data were unavailable or if they fell into the following categories: review articles, non-human studies, clinical guidelines, case reports, letters to the editor, editorials, conference posters, commentaries, and book chapters. Two independent reviewers conducted the screening process, including title, abstract, and full-text assessment to identify studies that met the predefined eligibility criteria. Any discrepancies between reviewers were resolved through discussion. The selection process and reasons for exclusion were documented using the PRISMA flow diagram. No restrictions were applied regarding patients’ age, sex, ethnicity, or geographic location.

### Searching Strategy

A comprehensive systematic literature search was conducted from database inception through 18 May 2025 across the following electronic databases: PubMed, Google Scholar, Web of Science (ISI), Scopus, SIGLE, Virtual Health Library (VHL), New York Academy of Medicine (NYAM), ClinicalTrials.gov, metaRegister of Controlled Trials (mRCT), EMBASE, Cochrane Library, and the World Health Organization International Clinical Trials Registry Platform (WHO ICTRP). No restrictions were imposed regarding participants’ age, sex, ethnicity, language, race, or geographic location. The search strategy utilized controlled vocabulary specific to each database’s thesaurus, employing a combination of Medical Subject Headings (MeSH) and free-text terms to maximize sensitivity and comprehensiveness. In addition, a manual search of the reference lists of all included studies was performed to identify further eligible articles not captured in the initial search. This cross-referencing process was continued iteratively until no additional relevant studies were found. The search strategy incorporated the following key terms in various combinations: “Radiotherapy,” “Radiation,” “DIEP,” “Deep Inferior Epigastric,” “Breast,” “Mammary,” and “Reconstruction.”

### Data Extraction

Data extraction was independently performed by two reviewers using a pre-structured Microsoft Excel spreadsheet. The following study-level characteristics were extracted from the included articles: study title, surname of the first author, year of publication, study design, study duration, and geographical location. Methodological variables were also recorded, including radiotherapy dosage, radiation protocol, study endpoints, and duration of follow-up. Patient baseline demographic data were collected, encompassing sample size, number of flaps, age, body mass index (BMI), and relevant comorbidities. Breast cancer-specific information was extracted, such as cancer type and stage, type of mastectomy performed, weight of the inset DIEP flap, and use of adjuvant chemotherapy or hormonal therapy. Surgical and aesthetic outcome measures included mean change in flap volume, total flap loss, partial flap necrosis, fat necrosis, flap contracture, need for revisions of the reconstructed breast, recipient site infections, wound-related complications, and patient satisfaction using BREAST-Q scores.

### Quality Assessment

The quality of the observational studies was estimated using the National Institute of Health (NIH) quality assessment tool [16]. The studies were assorted into good, fair, and bad when the score was <65%, 30–65%, and> 30%, respectively.

### Timing of Radiotherapy

Patients were stratified according to the timing of radiotherapy in relation to reconstruction. Pre-DIEP radiotherapy was defined as irradiation of the chest wall administered after mastectomy and prior to DIEP flap reconstruction, whereas post-DIEP radiotherapy referred to irradiation delivered to the reconstructed breast following completion of the DIEP procedure. Outcomes were subsequently compared between both cohorts to determine the effect of radiotherapy timing on reconstructive results.

### Statistical Analysis

Continuous outcomes were synthesized using either the standardized mean difference (SMD) or weighted mean difference (WMD), as appropriate. When data were reported as mean and range or median and range, they were converted to mean and standard deviation (SD) following established statistical methods [17]. For dichotomous outcomes, pooled estimates were calculated using the risk ratio (RR) with corresponding 95% confidence intervals (CIs). A fixed-effect model was applied when homogeneity across studies was assumed; otherwise, a random-effects model was employed to account for potential between-study variability. Statistical heterogeneity was assessed using Higgins’ *I*^2^ statistic, with a threshold of >50% indicating substantial heterogeneity, and the Cochrane Q test (Chi^2^), with a significance level set at *p* < 0.10 [18]. Subgroup analyses were conducted based on the timing of radiotherapy. Sensitivity analyses were performed to evaluate the robustness of the results by including only patients who received post-DIEP radiotherapy. All statistical analyses were performed using Review Manager software (RevMan, version 5.4) [19]. A *p* value <0.05 was considered statistically significant.

## Results

**Fig. 1:**
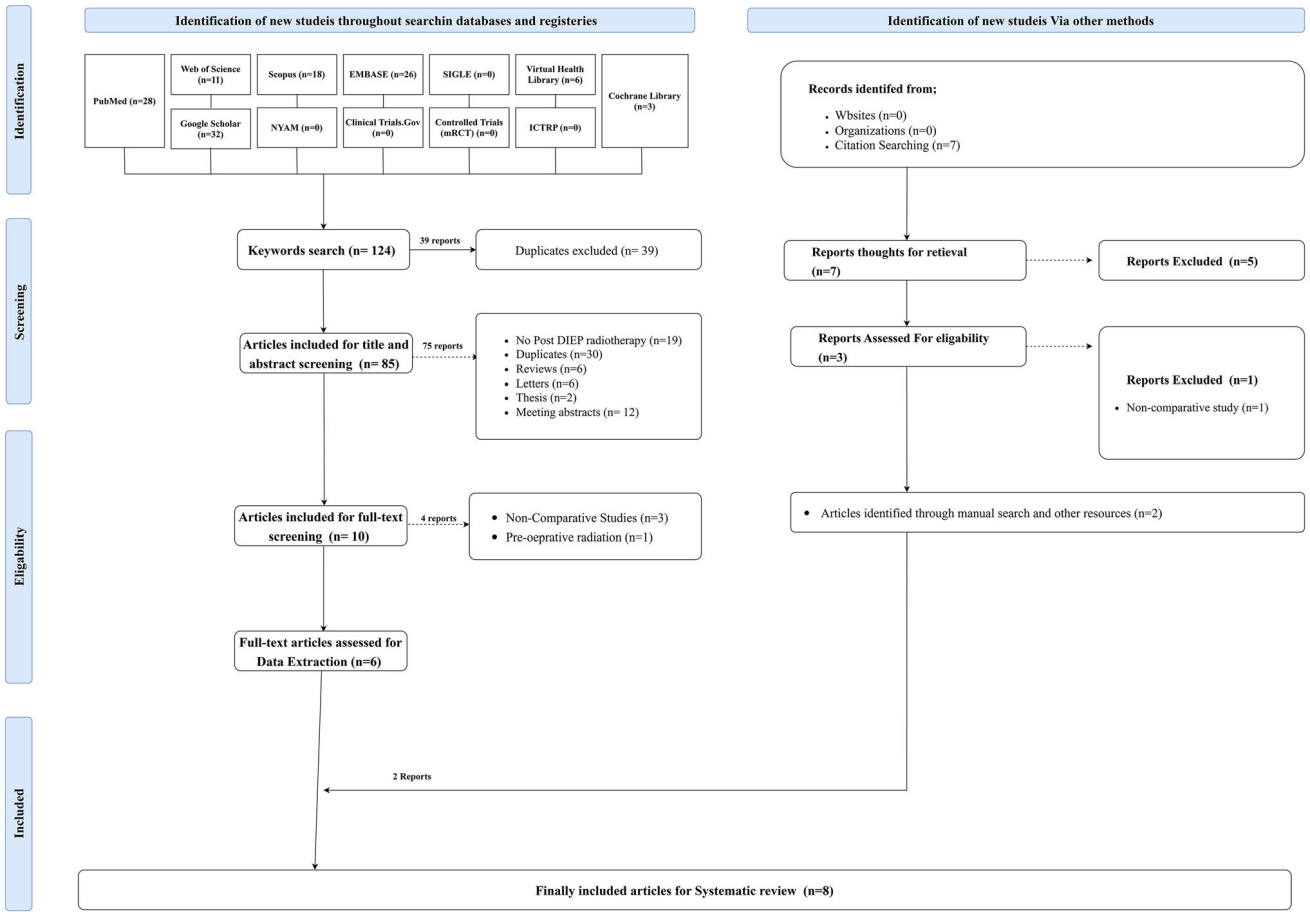
PRISMA flowchart showing the process of the literature search, title, abstract, and full text screening, systematic review, and meta-analysis

A comprehensive literature search initially identified 124 articles. After the exclusion of 39 duplicate studies, 85 articles remained for title and abstract screening. Seventy-five reports were excluded, and ten articles were included for full-text screening. Four articles were excluded, and two were identified through citation tracking, resulting in eight articles that were finally included in the systematic review and meta-analysis (Fig. 1).

### Demographic Characteristics of the Included Studies

The present study included eight articles [20–27] encompassing 4467 patients with 4478 DIEP flaps for breast reconstruction. There were six articles of prospective design and two retrospective studies. Seven studies investigated immediate DIEP flap reconstruction. Except for Craig et al. [21], all studies utilized between-patient comparisons, comparing outcomes across distinct patient groups rather than within the same individual. Of the patients included, 1645 received radiation therapy. The average irradiation dosage hovered between 40 Gy and 6120 cGy. The average age of the patients ranged from 34 to 54.9 years among the irradiated group and from 35 to 53 years among the non-irradiated group. There were 496 smokers, and the average BMI ranged from 24.2 to 30.2 kg/m^2^. Of the included patients, 2736 patients received chemotherapy, while 124 received hormonal therapy. The initial flap volume ranged from 580 to 760 ml among the irradiated group and from 513 to 682.2 ml among the non-irradiated group. The average follow-up period ranged from three months to 5.73 years. All of the observational studies analyzed were of good quality (Table 1).

**Table 1:** Demographic characteristics of the included studies

Study ID		Study region	Study design	Study period	Radiation dosage	Timing of radiotherapy	Timing of reconstruction	Comparison group	Sample Size		Number of flaps		Age (Years)	
									Radiotherapy	Control	Radiotherapy	Control	Radiotherapy	Control
									Number	Number	Number	Number	Mean (SD)	Mean(SD)
1	Chatterjee et al. [20]	UK	Prospective	1995 to 2005	45 Gy in 20 fractions over 4 weeks	Post DIEP	Immediate	Between patients	22	46	NR	NR	51 (37–66)*	53(38–70)*
2	Craig et al. [21]	USA	Prospective	2005 and 2014	NR	Post DIEP	Immediate	Within Patients	11	11	NR	NR	NR	NR
3	Hsieh et al. [22]	Taiwan	Retrospective	July 2008 and December 2018	5000–5040 cGy divided into 25–28 fractions	Post DIEP	Immediate	Between patients	15	86	NR	NR	40± 7.5	45.5 ± 8.3
4	Lukas et al. [24]	Germany	Prospective	January 2011 and January 2019	NR	Pre DIEP	Immediate	Between patients	1447	2479	1642	2935	51.8±33	51.1±30.8
5	Nava et al. [27]	Switzerland	Retrospective	January 2018 and June 2024	50.5 ± 10.8	Pre DIEP	immediate or delayed	Between patients	21	29	NR	NR	49±6.3	54.3±11.1
6	O'Connell et al. [23]	UK	Prospective	1, 2009, and October 1, 2014	40 Gy in 15 fractions over 3 weeks	Post DIEP	Immediate	Between patients	28	80	NR	NR	54.9 (48.6–60.7)**	52.8 (49–60.3)**
	O'Connell et al. [23]					Pre-DIEP	Delayed	Between patients	21	80	NR	NR	46.6 (43.5–50.4)**	
7	Rogers et al. [25]	USA	Prospective	January 1994 and November 1999	4400 to 6120	Post DIEP	Immediate	Between patients	30	30	NR	NR	34–68	35–62
8	Taghizadeh et al. [26]	UK	Prospective	NR	NR	Post DIEP	Immediate	Between patients	50	62	61	95	47.5	47.1

BMI (Kg/m^2^)		Smokers		Adjunctive therapy				Initial Flap Volume (ml)		Follow-up (Years)		Quality assessment	
				Chemotherapy		Hormonal Therapy							
Radiotherapy	Control	Radiotherapy	Control	Radiotherapy	Control	Radiotherapy	Control	Radiotherapy	Control	Radiotherapy	Control		
Mean(SD)	Mean(SD)	Number	Number	Number	Number	Number	Number	Number	Number	Mean(SD)	Mean(SD)	%	Decision
NR	NR	NR	NR	12	13	18	26	760 (380–1400)*	610 (260–1600)*	3.5 (1–10) years*		66.66%	Good
NR	NR	NR	NR	NR	NR	NR	NR	NR	NR	2.53 years		66.66%	Good
24.5 ± 3.7	24.2 ± 4.2	NR	NR	21	62	NR	NR	NR	NR	5.2 ±2.48	5.73 ±2.73	66.66%	Good
26.7±4.6	26±4.4	175	301	1322	1283	NR	NR	NR	NR	Three months		66.66%	Good
25.9±4.6	26.1±4.7	4	9	NR	NR	NR	NR	NR	NR	16 Months		75%	Good
27.6 (23.5–29.3)*	24.6 (22.5–28.6)*	0	4	22	19	23	40	580 (428–762)**	513 (400–685)**	NR	NR	66.66%	Good
25.6 (22.6–28.6)		0		19		17		585 (444–750)**					
26.1	26.2	5	2	NR	NR	NR	NR	NR	NR	19.9 months	17.4 months	66.66%	Good
29.4	30.2	4	5	31	15	NR	NR	672.8(336, 1447)*	682.2 ((320, 1390)*	33.3 months	32.9 months	66.66%	Good

*DIEP* Deep inferior epigastric artery perforator flap, *BMI* Body mass index, *Data reported using median and range

**Data reported in the form of median and interquartile range

### The Impact of Radiotherapy on the Outcomes of DIEP Flap

#### Flap Volume

**Fig. 2:**
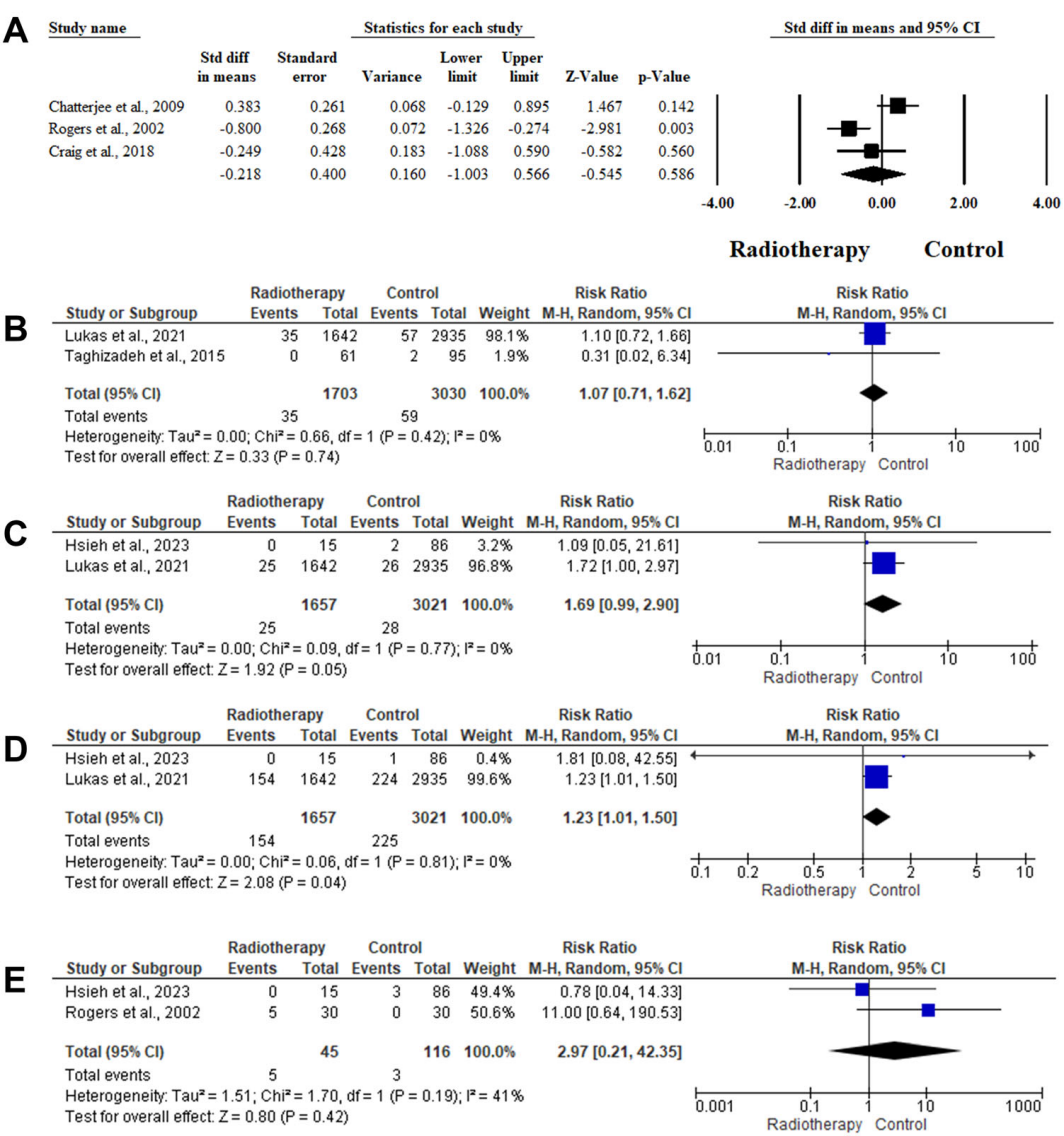
Forest plot of summary analysis and 95% CI of **A** Standardized mean difference in mean change in flap volume between the irradiated and the non-irradiated breasts. **B** The risk of total flap losss between the irradiated and the non-irradiated breasts. **C** The risk of partial flap loss between the irradiated and the non-irradiated breasts. **D** The risk of wound revisions between the irradiated and the non-irradiated breasts. **E** The risk flap contracture between the irradiated and the non-irradiated breasts. Size of the blue squares is proportional to the statistical weight of each trial. The black diamond represents the pooled point estimates. The positioning of both diamonds and squares (along with 95% CIs) beyond the vertical line (unit value) suggests a significant outcome (*IV* inverse variance)

Three studies including 150 flaps evaluated the change in flap volume between the irradiated and the non-irradiated breasts after DIEP flap for breast reconstruction [20, 21, 25]. There was no statistically significant difference (*P* = 0.586) between the irradiated and the non-irradiated breasts regarding the mean change in flap volume (SMD; − 0.218, 95% CI − 1.003) after pooling the data in the random-effects model (*I*^2^=79.99%, *P* = 0.007) (Fig. 2A).

#### Total Flap Loss

Two articles included 4733 flaps evaluated the risk of total flap necrosis between the irradiated and the non-irradiated breasts after DIEP flap for breast reconstruction [24, 26]. The risk of total flap necrosis after radiotherapy was 1.07 (95% CI 0.71, 1.62, *P* = 0.74) in the random-effects model (*I*^2^=0%, *P* = 0.42) (Fig. 2B).

#### Partial Flap Loss

The risk of partial flap necrosis was evaluated among 4678 flaps within two articles [22, 24]. The risk of partial flap loss was 1.69 among irradiated breasts (95% CI 0.99, 2.90, *P* = 0.05) in the random-effects model (*I*^2^=0%, *P* = 0.77) (Fig. 2C).

#### Wound Revisions

Two articles included 4678 flaps evaluated the risk of wound revisions after DIEP flap for breast reconstruction [22, 24]. The risk of wound revisions was 1.23 among irradiated breasts, with 95% CI hovering between 1.01 and 1.50 (*P* = 0.04) in the random-effects model (*I*^2^=0%, *P* = 0.81) (Fig. 2D).

#### Flap Contracture

The risk of flap contracture was evaluated among 161 DIEP flaps for breast reconstruction [22, 25]. The risk of flap contracture was 2.97 times higher among patients with post-DIEP irradiated breasts (95% CI 0.21, 42.35, *P* = 0.42) after pooling the data in the random-effects model (*I*^2^=41%, *P* = 0.19) (Fig. 2E).

#### Fat Necrosis

**Fig. 3:**
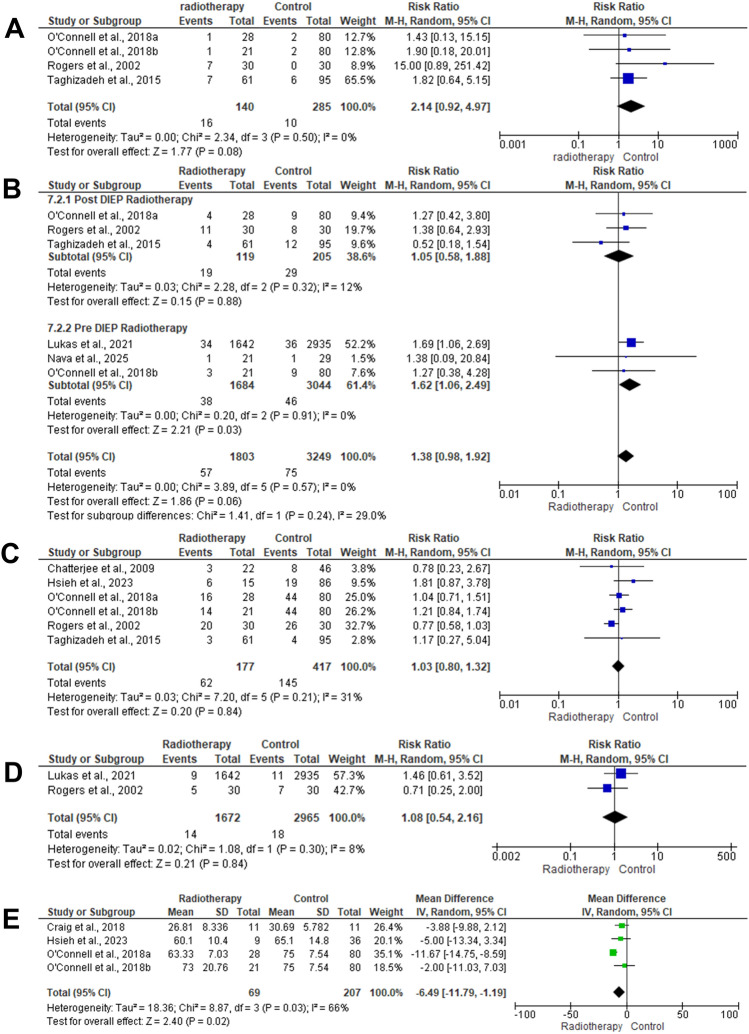
Forest plot of summary analysis and 95% CI of **A** The risk of fat necrosis between the irradiated and the non-irradiated breasts. **B** The risk of wound healing wound disturbances between the irradiated and the non-irradiated breasts. **C** The risk of reconstructed breast adjustments between the irradiated and the non-irradiated breasts. **D** The risk of recipient site infection between the irradiated and the non-irradiated breasts. **E** The mean difference in total satisfaction with breasts between the irradiated and the non-irradiated breasts. Size of the blue squares is proportional to the statistical weight of each trial. The black diamond represents the pooled point estimates. The positioning of both diamonds and squares (along with 95% CIs) beyond the vertical line (unit value) suggests a significant outcome (*IV* inverse variance)

Three articles included 425 flaps evaluated the impact of radiotherapy on the risk of fat necrosis among patients with DIEP flaps for breast reconstruction [23, 25, 26]. The risk of fat necrosis was 2.14 times higher among the irradiated breasts in comparison to non-irradiated breasts (RR; 2.14, 95% CI 0.92, 4.97, *P* = 0.08) in the random-effects model (*I*^2^=0%, *P* = 0.5). A sensitivity analysis including only patients who received post-DIEP radiotherapy revealed a risk of fat necrosis of 2.32 times (95% CI 0.78, 6.93; *P* = 0.13). **(**Fig. 3A**)**

### Wound Healing Disturbances

Five articles included 5052 flaps evaluated the risk of wound healing wound disturbances after radiotherapy among patients with DIEP flap for breast reconstruction [23–27]. Pooling the data in the random-effects model (*I*^2^=0%, *P* = 0.57) revealed no statistically significant difference between irradiated and non-irradiated breasts (RR; 1.38, 95% CI 0.98, 1.92, *P* = 0.07). Subgroup analysis based on the timing of radiotherapy revealed a statistically significant higher risk of wound healing disturbances associated with pre-DIEP radiotherapy (*P* = 0.03) with an RR of 1.62 (95% CI 1.06, 2.49) (Fig. 3B).

### Reconstructed Breast Adjustments

Five articles included 594 flaps evaluated the need for adjustments after DIEP flap for breast reconstruction among the irradiated and the non-irradiated breasts [20, 22, 23, 25, 26]. In the random-effects model (*I*^2^=31%, *P* = 0.21), there was no statistically significant difference between both groups (RR 1.03, 95% CI 0.8, 1.32, *P* = 0.84). A sensitivity analysis including only patients who received post-DIEP radiotherapy revealed no statistically significant difference between irradiated and non-irradiated groups (RR; 0.97, 95% CI 0.72, 1.32, *P* = 0.86) (Fig. 3C).

### Recipient Site Infection

Two articles included 4637 flaps evaluated the risk of recipient site infection among patients who received radiotherapy after DIEP flap for breast reconstruction [24, 25]. There was no statistically significant difference between the irradiated and the non-irradiated breasts (*P* = 0.84) with a RR of 1.08 hovered between 0.54 and 2.16 in the random-effects model (*I*^2^=8%, *P* = 0.30) (Fig. 3D).

### Total Breast Satisfaction Score

Three articles included 276 patients evaluated the difference in total breast satisfaction using BREAST-Q scores between irradiated and non-irradiated breasts after DIEP reconstruction [21–23]. In the random-effects model (*I*^2^=66%, *P* = 0.03), there was a statistically significant lower total breast satisfaction score among irradiated breasts with an MD of − 6.49 (95% CI − 11.79, − 1.19, *P* = 0.02). A sensitivity analysis including only patients who received post-DIEP radiotherapy revealed a statistically significant lower total breast satisfaction score compared with non-irradiated breasts (MD − 7.53; 95% CI − 13.30 to − 1.77; *P* = 0.01). (Fig. 3E**)**

## Discussion

Radiotherapy has a crucial role in controlling locoregional recurrence and improving survival in patients with breast cancer. The desire to offer aesthetically appealing and safe oncological breast reconstruction highlights the need to evaluate the impact of radiotherapy on the outcomes of DIEP flaps for breast reconstruction [28]. The present meta-analysis revealed a relatively higher risk of complications and poor satisfaction with breasts among patients subjected to radiotherapy and DIEP flap. Radiotherapy increases the risk of partial flap loss, wound revisions, fat necrosis, and flap contracture, along with a remarkable decline in overall satisfaction with breasts. Pre-DIEP radiotherapy was associated with a higher risk of wound healing disturbances. Despite the higher risk of partial flap loss, fat necrosis, and flap contracture, it does not achieve a statistically significant result. Although there was an increased risk of secondary surgery to address clinical complications in the irradiated groups, there was no significant difference in the rates of revisional surgery for cosmetic improvement. There was no significant difference between the irradiated and the non-irradiated breasts regarding the mean change in flap volume, the number of reconstructed breast adjustments, and recipient site infections. Sensitivity analyses restricted to patients receiving post-DIEP radiotherapy demonstrated a statistically significant reduction in total breast satisfaction, consistent with the overall results. Although trends toward increased rates of fat necrosis and other complications were observed, these did not attain statistical significance, likely reflecting the limited sample size.

The present study revealed a relatively higher risk of wound healing disturbances and wound revisions among irradiated breasts. Radiotherapy increases local collagen deposition and disrupts angiogenesis in the vascular bed, reducing perfusion and extensive parenchymal changes in the reconstructed breasts [29]. Radiotherapy induces oxidative stress, endothelial inflammation, DNA damage, and fibrosis within the stroma of fat tissue. This results in cell death, fat necrosis, and wound healing disturbances [30, 31]. In this respect, reported a higher risk of fat necrosis following post-reconstruction radiotherapy compared with pre-reconstruction and a much lower risk among patients with no history of radiotherapy in autologous abdominal-based breast reconstruction [32]. Liew et al. [11] reported a relatively higher risk of fat necrosis, secondary surgery, and volume loss among irradiated breasts after autologous breast reconstruction yet with comparable risks of clinical outcomes and overall satisfaction in the irradiated and non-irradiated flaps. Schaverien et al. [33] reported satisfactory outcomes of radiation therapy and immediate breast reconstruction, apart from an increased risk of revisional surgery among patients with immediate rather than delayed breast reconstruction. Contrary to our findings, Kelley et al. [34] reported no significant difference in surgical and clinical outcomes before and after radiotherapy with autologous breast reconstruction. Ward et al. [35] demonstrated that preoperative radiotherapy achieved oncological outcomes and surgical complications within normal limits. Cabañuz et al. [36] reported a higher risk of reconstruction failure and reoperations among patients who underwent radiotherapy before breast reconstruction.

Despite the increased risk of wound revisions among the irradiated breasts, the number of reconstructed breast adjustments for cosmetic improvement was similar among the irradiated and non-irradiated breasts. In this respect, there was no significant difference in mean change in flap volume between the irradiated and the non-irradiated breasts. Optimizing the volumetry of the DIEP flap in the settings of radiotherapy may minimize the need for subsequent breast adjustments after the completion of radiation therapy [37]. The appropriate degree of overcorrection when designing DIEP flaps in the setting of radiotherapy is crucial. Myung et al. [38] recommended an overcorrection of 14% in reconstruction flap volume after observing an approximate 12% decline in breast volume after radiotherapy. Improved vascularization of the flaps renders them radioresistant, reducing the risk of radiotherapy-associated adverse events [39]. Yiwen et al. [40] revealed that preoperative planning, perioperative monitoring, and post-operative care could significantly minimize the risk of DIEP flap necrosis in patients with breast reconstruction.

Post-mastectomy radiation therapy was associated with poor satisfaction with breasts after DIEP flaps. Zugasti et al. [5] reported poor aesthetic outcomes and lower patient-reported satisfaction in the settings of post-mastectomy radiation therapy both in implant-based and autologous-based breast reconstruction. The damage effect of radiation therapy on the reconstructed breasts with DIEP flaps may be associated with poor patients’ satisfaction, particularly when compared with pre-radiation status [41]. These findings highlighted the need to maximize patient satisfaction by conveying an individualized approach and managing expectations for patients undergoing DIEP flaps for breast reconstruction. A multidisciplinary approach should be tailored for each patient to minimize complications while maximizing satisfaction and aesthetic outcomes. The present systematic review revealed the impact of radiotherapy on the outcomes of DIEP flap for breast reconstruction. The results of this review should be cautiously interpreted with the presence of some limitations. There was remarkable heterogeneity in study designs, radiation protocols, reconstructive patterns, outcome measures, and follow-up periods. The effect of radiotherapy on the DIEP flap for breast reconstruction in the long term may not be predictable, with a degradation of cosmetic results over time [42, 43]. The random-effects model and subgroup analysis were performed to address the heterogeneity between the analyzed outcomes.

## Conclusions

Radiotherapy adversely affects surgical and patient-reported outcomes following DIEP flap autologous breast reconstruction, with pre-DIEP radiotherapy significantly increasing the risk of wound healing disturbances and post-DIEP radiotherapy associated with reduced overall breast satisfaction. Identifying such evidence could help to individualize the appropriate radiotherapy regimen and reconstructive approach, along with implementing refinement procedures to lessen the negative consequences of radiotherapy on the DIEP flap for breast reconstruction.

## Supplementary Information

Below is the link to the electronic supplementary material.

Supplementary file1 (DOCX 30 KB)

## Data Availability

The datasets used in the present study are available from the first author and corresponding authors on reasonable request.
